# Infection Control in Small Residential Care Settings: Insights From a National Survey and Washington State Data

**DOI:** 10.1093/geroni/igab046.3499

**Published:** 2021-12-17

**Authors:** Carolyn Ham, Anna Unutzer

**Affiliations:** 1 Washington State Department of Health, Olympia, Washington, United States; 2 Washington State Department of Health, Shoreline, Washington, United States

## Abstract

Infection control is a vital issue in long-term care, and the increasing popularity of small residential care facilities (SRCF) raises questions about the effectiveness of this model for preventing facility-acquired infections. In SRCF, care is provided in a residential home to a small number of residents. The setting lacks common terminology, and states license SRCF under various titles including Adult Family Homes, Adult Foster Homes and Family Care Homes. To better inform infection control efforts in this unique setting type, DOH staff conducted a comprehensive search to locate states that license SRCF. A total of 24 states were identified and approached to participate in a qualitative research study; 21 responded, three declined and nine were unable to participate due to staff time constraints. Between March 12th and April 15th, 2021, ten public health and regulatory staff from nine states completed semi-structured telephonic interviews on infection control in SRCF. Infection control licensing requirements and public health oversight for SRCF varied significantly across participating states. Data from these interviews was analyzed and compared with two Washington State Adult Family Home (AFH) sources: 1) online survey of AFH providers 2) Infection Control Assessment and Response evaluations conducted by public health staff. Four themes were identified in all three data sets: access to personal protective equipment, environmental safety, staffing issues and knowledge deficits. SRCF are valued by states that license them. Despite the challenges of implementing infection control in the home-like environment, extraordinary opportunities exist for improving care and preventing infections in this setting.

